# Luminescence Thermometry with Nanoparticles: A Review

**DOI:** 10.3390/nano13212904

**Published:** 2023-11-05

**Authors:** Ljubica Đačanin Far, Miroslav D. Dramićanin

**Affiliations:** Centre of Excellence for Photoconversion, Vinča Institute of Nuclear Sciences—National Institute of the Republic of Serbia, University of Belgrade, P.O. Box 522, 11001 Belgrade, Serbia; ljubica.far@vin.bg.ac.rs

**Keywords:** luminescence thermometry, luminescence, nanoparticles, thermometry, nanophosphors, quantum dots, nanodiamonds

## Abstract

Luminescence thermometry has emerged as a very versatile optical technique for remote temperature measurements, exhibiting a wide range of applicability spanning from cryogenic temperatures to 2000 K. This technology has found extensive utilization across many disciplines. In the last thirty years, there has been significant growth in the field of luminous thermometry. This growth has been accompanied by the development of temperature read-out procedures, the creation of luminescent materials for very sensitive temperature probes, and advancements in theoretical understanding. This review article primarily centers on luminescent nanoparticles employed in the field of luminescence thermometry. In this paper, we provide a comprehensive survey of the recent literature pertaining to the utilization of lanthanide and transition metal nanophosphors, semiconductor quantum dots, polymer nanoparticles, carbon dots, and nanodiamonds for luminescence thermometry. In addition, we engage in a discussion regarding the benefits and limitations of nanoparticles in comparison with conventional, microsized probes for their application in luminescent thermometry.

## 1. Introduction

Nanoparticles are defined as materials that possess dimensions inside the nanoscale range, namely measuring less than 100 nm. These materials have become important players in contemporary technologies, with numerous and significant uses. Luminescent nanoparticles have garnered substantial attention among the scientific community due to their many present-day uses, including but not limited to solid-state lighting, solar cells, displays, dosimetry, medical imaging, phototherapy, counterfeiting, and physical and chemical sensing [1]. Nanoparticles exhibit characteristics that are not seen in their bulk counterparts as a result of the reduction in their size. The electronic and phonon quantum confinements, high surface area, and surface features facilitating specialized interactions are key factors contributing to their distinct properties and appealing applications.

Luminescent nanoparticles are also successfully used as luminescence thermometry probes. This optical technique for the remote sensing and measurement of temperature relies on the detection of temperature-induced changes in the luminescence of probe materials. The detection can be steady-state or time-resolved in both downshifting and up-conversion emission measurements. So far, all types of luminescent materials have been tested for various applications of luminescence thermometry. This is possible because luminescence is always affected by temperature to some extent. The type of material used depends on application demands and available instrumentation. However, luminescent nanoparticles were the sole choice for luminescence thermometry in biomedicine, integrated optoelectronics, and nanoscale environments [2]. They were used either just for temperature sensing or to simultaneously serve additional tasks, such as bioimaging, localized heating, chemical sensing, etc. The method can be used in biological systems in vitro or in vivo, where temperature variations in kelvin have been measured. This amount of temperature variation cannot be justified considering the power generated by cell metabolism and the laws of thermal transport [3]. The quantum efficiency of emission and the biocompatibility of the material are also important factors to consider when utilizing nanoparticles.

The spectral positions of emission and excitation bands, the intensity of emission bands, the ratio of the intensities of emission bands, excited state lifetimes, and emission rise times are the features readily used to determine the temperature of luminescence probes. They can be used either separately or in combination to perform single-parameter thermometry or multiparameter thermometry. In this case, the choice depends not only on the application demands and available instrumentation but also on the available materials. The latter differ in luminescence characteristics and in thermometric performances, which are usually quantified by the relative sensitivity, temperature, and spatial and temporal resolutions. It is worth mentioning that the thermometric performances of luminescence nanoparticles differ from those of their bulk counterparts [4]. In addition, some of them, mostly semiconductor quantum dots, exhibit notable size dependence.

A substantial body of literature exists on the subject of luminescence thermometry, encompassing several books [2,4,5] and review papers [6,7,8,9,10]. Nevertheless, there is a notable absence of comprehensive review sources that thoroughly explore the application of nanoparticles in luminescent thermometry. This review paper aims to address this deficiency by providing a comprehensive analysis of this area. It is primarily focused on an overview of the recent utilization of nanoparticles for luminescence thermometry and not on the luminescence thermometry methodology itself. The review addresses both inorganic and organic nanoparticles, namely lanthanide and transition metal nanophosphors, semiconductor quantum dots, polymer nanoparticles, carbon dots, and nanodiamonds.

## 2. Lanthanide Activated Nanoparticles

**Figure 1:**
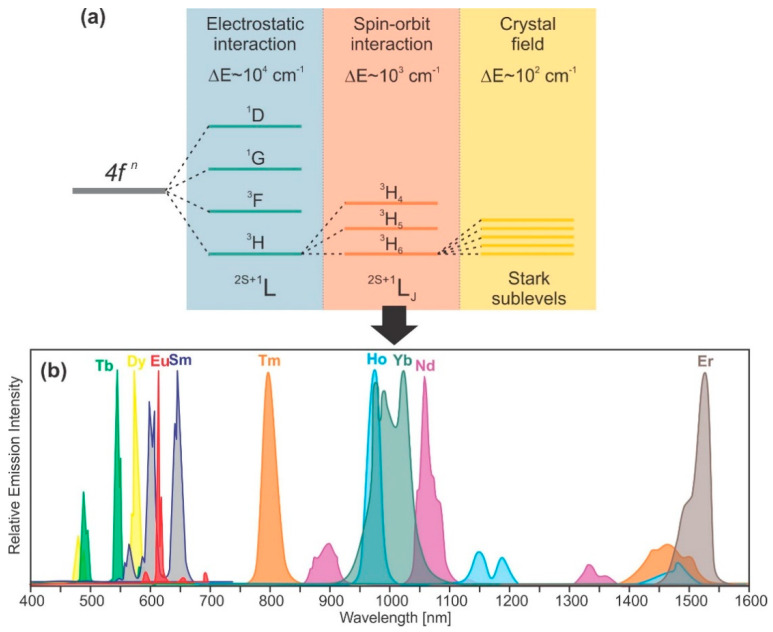
(**a**) Ln^3+^ ion’s energy levels splitting; (**b**) Emission spectra of different Ln^3+^ ions between 400 and 1600 nm.

Lanthanide-activated materials were among the first to find usage in luminescence-based temperature measurements and have been, by far, the most widely employed [4]. They consist of lanthanide ions as optically active centers that are incorporated into hosting insulators or wide band gap semiconductors. Even though lanthanides include sixth group elements form the periodic table, from ^57^La to ^71^Lu, only elements from ^58^Ce to ^70^Yb transform into ions that possess specific electronic configurations which result in a diversity of luminescence features. The most common trivalent lanthanide ions are formed by removing electrons from the outer *5d* and/or *6s* orbitals, leaving only partly filled *4f* orbitals. In this way, optically active *4f* electrons are shielded inside the outer *5s^2^* and *5p^6^* shells, which are in fact energetically lower. This indicates that the optical properties of lanthanides are only slightly impacted by their surroundings, resulting in typically narrow spectral bands formed by sets of well-defined peaks. However, the crystal environment is important in lanthanide luminescence because electronic transitions between 4f levels, which are forbidden by Laporte’s selection rules, become partially allowed when the lanthanide senses an asymmetric field from the host crystal lattice [11]. The diversity of energy levels of a lanthanide ion within the crystal lattice is depicted schematically in Figure 1a, including the order of magnitude of the energetic separation between sublevels. The levels are noted as LJ2S+1, (L is orbital angular momentum, noted as S, P, D, F, G, H, … for L=1, 2, 3, 4, 5, 6…, respectively; S is spin angular momentum; J is total angular momentum; J=L−S when n<7 and J=L+S when n>7), and are much more influenced by the spin–orbit interaction than the crystal field. The number of Stark sublevels that originated from the crystal field is 2J+1 when J is an integer and J+1/2 when J is a half-integer number. Numerous energy levels of lanthanide ions provide a variety of luminescent emissions throughout the long spectral range from UVA to NIR, Figure 1.

Each lanthanide ion has a specific luminescent fingerprint according to its set of energy states [11]. Trivalent Sm^3+^ (*4f*^5^ configuration), Eu^3+^ (*4f*^6^), Tb^3+^ (*4f*^8^), Dy^3+^ (*4f*^9^), and Ho^3+^ (*4f*^10^) ions have largely separated energy levels and exhibit strong emissions in the visible region [4]. Nd^3+^ (*4f*^3^), Er^3+^ (*4f*^11^) and Tm^3+^ (*4f*^12^) ions, whose energy levels have smaller energy differences, usually radiate in the NIR region. The Ce^3+^ ion is an unusual example of trivalent lanthanides because of its large energy *4f*–*5d* transition, which differs from the typical *4f*–*4f* lines of other Ln^3+^. The emission of Gd^3+^ is located at a shorter wavelength in the ultraviolet spectral range due to the absence of sufficient intermediate states; therefore, it is not commonly used as an emitting center in luminescence thermometry [4]. Only recently, Yu et al. [12] have demonstrated that YAl_3_(BO_3_)_4_ is activated by the Pr^3+^–Gd^3+^ pair for ratiometric luminescence thermometry in the UV spectral range.

Regardless of the excitation scheme, the luminescent properties of lanthanide-doped nanoparticles are temperature-dependent in a variety of ways. As the temperature rises, the various emission peaks broaden as a result of lattice vibrations, the overall intensity of the spectrum decreases, and the lifetimes of radiative transitions shorten as non-radiative phonon relaxations become more probable. All of these phenomena have been proposed and verified as a basis for temperature sensing with lanthanide-doped nanoparticles [4,6,13]. Each lanthanide energy state can potentially be used to sense temperature. Depending on the experiment, this provides a wide spectrum of excitation and emission wavelengths [14]. As primary disadvantages, one should mention the lower absorption cross-sections and quantum yields of *f–f* transitions in lanthanide-doped nanoparticles compared with those of other luminescent probes, which must often be compensated for by a higher excitation power or a prolonged detection time [14]. The ability of this class of nanothermometers to optimize sensing performance for different temperature ranges—from cryogenic regions (4 K) to regions with temperatures as high as >1400 K—is a remarkable advantage for many applications including those in harsh environments [15,16,17]. From the point of view of biocompatibility, while lanthanide doped NPs are basically considered as chemically nontoxic, toxicity due to nanoparticle size may pose some concerns [18].

The initial utilization of lanthanide-based phosphors for luminous temperature monitoring was documented by Kusama et al. [19], who investigated the cathodoluminescence of Y_2_O_2_S:Eu^3+^. Interest in this topic surged around 2010, owing mostly to the realization of luminescence thermometry’s great potential in nanotechnology and nanomedicine [6].

Lanthanide-based nanoparticles (NPs) utilized in luminescent thermometry can be categorized into two types, based on energy path phenomena and the number of photons absorbed and released. These types are known as *downshifting* and *upconverting* NPs [20]. In downshifting, NPs absorb high-energy photons and subsequently re-emit lower energy photons. Almost all lanthanide ions can be used for Boltzmann-type LIR temperature sensing, and downshifting lanthanide-based NPs are a good choice when a high energy absorption is practically feasible [4]. Most literature reports involve the use of Eu^3+^, Dy^3+^ and Nd^3+^ ions.

**Figure 2:**
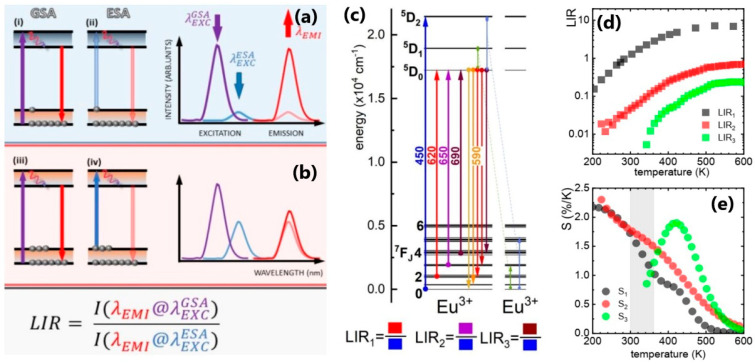
Schematic presentation of excited state absorption-based ratiometric luminescent thermometry at low (**a**) and high (**b**) temperatures; (**c**) Simplified energy diagram of Eu^3+^ ions with the indication of level used for temperature sensing; (**d**,**e**) The results of LIR and temperature sensitivity. Adapted with permission from [21].

Recently, Trejgis et al. [21] developed a new concept of luminescence-based temperature sensing and 2D mapping using the intensity of the emission band under excited state absorption (ESA) of Eu^3+^-doped LiLaP_4_O_12_ nanocrystals of approximately 20 nm size. In this particular approach, it is shown that at lower temperatures, the population of the ground state is predominant (Figure 2a). Consequently, the absorption process of the ground state becomes highly effective, resulting in a strong intensity of the excited emission achieved at the wavelength $\lambda_{EMI}^{GSA}$ (wavelength of the emission obtained upon the ground state absorption). Nevertheless, as per the Boltzmann distribution, it is seen that at higher temperatures, the relative population of the excited state (^7^F_1–6_) is greater than that of the ground state (^7^F_0_), Figure 2b. In this particular scenario, the temperature-dependent factor pertains to the LIR (Luminescence Intensity Ratio) of a solitary emission band acquired using two distinct excitation wavelengths, one resonating with the Ground State Absorption (GSA) and the other resonating with the Excited State Absorption (ESA). The authors examined the excitation and the emission within the temperature range of −150 to 400 °C and obtained sensitivity as high as 2.17% K^−1^ (at 200 K), and 1.9% K^−1^ (at 200 K).

In Table 1, the most recent results of typical nanothermometry studies of downshifting nanoparticles made from lanthanides are shown.

*Upconverting* nanoparticles (UC NPs) possess the potential to achieve light emission characterized by a shorter wavelength and more energy compared with the excitation process. This phenomenon is facilitated by a nonlinear optical process known as upconversion [13,14,34,35,36]. Typically, the phenomenon is seen through the sequential absorption of two or more photons with low energy, subsequently resulting in the emission of a photon with higher energy. Various mechanisms have been put forth to elucidate the phenomenon of upconversion, including excited-state absorption, energy transfer upconversion, photon avalanche, cooperative sensitization upconversion, and energy migration upconversion [37]. In general, the process of upconverting nanoparticles (UCNPs) involves the introduction of a single ion, known as the activator. This activator ion, which may include ions such as Er^3+^, Ho^3+^, and Tm^3+^, is responsible for generating emissions through excited state absorption or energy transfer between ions of the same species. Frequently, the addition of two distinct ions is employed to enhance the upconversion quantum yield and absorption cross-section. This involves the introduction of an activator ion alongside a sensitizer ion, which possesses the ability to absorb energy and subsequently transmit it to the adjacent activator within the crystal lattice [4,34]. An exemplary instance of an upconverting ion pair that holds significant relevance in the field of thermometry is the Yb^3+^–Er^3+^ pair. In this system, Yb^3+^ ions exhibit absorption of radiation at approximately 980 nm, while the absorbed energy is subsequently transferred to Er^3+^ ions, leading to their excitation through a series of resonant transitions. In addition to this, it is worth noting that the energy level of the excited state of Yb^3+^ closely aligns with that of Ho^3+^ and Tm^3+^ excited states. This alignment facilitates efficient resonant energy transfer [4]. The utilization of upconverting luminous nanoparticles appears primarily in biological contexts, as the use of low-energy near-infrared (NIR) excitations does not pose any harm to living entities [38]. The first report of a UCNP-based nanothermometer for in vitro measurement was reported by Vetrone et al. who used PEI-coated NaYF_4_:Yb^3+^/Er^3+^ nanocrystals to measure the intracellular temperature of HeLa cells [39]. Tm^3+^ ion is also suitable for UC nanothermometry in bioapplications since it emits within the biological windows of transparency of tissues [40].

**Figure 3:**
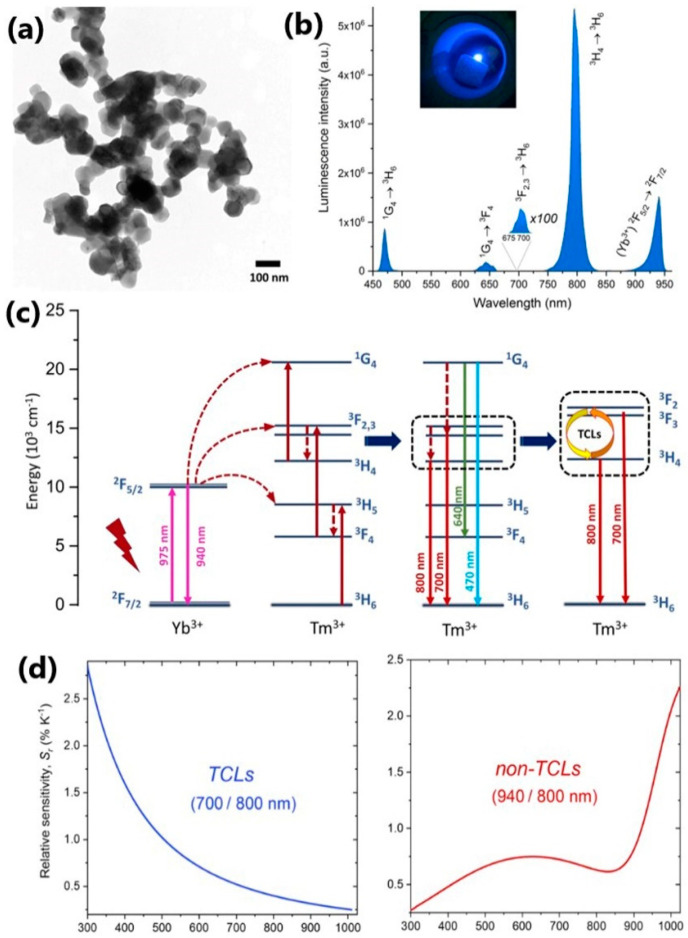
(**a**) TEM image; (**b**) UC emission spectrum; inset presents a photograph of the sample under 975 nm laser irradiation at ambient conditions; (**c**) a simplified energy-level diagram for the synthesized YVO_4_:Yb^3+^−Tm^3+^ NPs; (**d**) the obtained relative temperature sensitivities (Sr) for TCLs of Tm^3+^ (700/800 nm) and non-TCLs of Yb^3+^ and Tm^3+^ (940/800 nm) as a function of temperature. Adapted from [41] under a Creative Commons Attribution (CC-BY) License.

Runowski et al. [41] recently reported results of the upconverting nanoparticles of YVO_4_ co-doped with Yb^3+^-Tm^3+^ for sensing high temperatures—up to 1000 K. Their particles of 50–100 nm sizes exhibit upconversion emission of Tm^3+^ and Yb^3+^ ions upon the 975 nm laser excitation. For temperature sensing in the low-temperature range, the authors used relative intensities of the Tm^3+^ emissions from thermally coupled levels (TCLs), while for the high-temperature range, the intensity ratio of the nonthermally coupled levels (non-TCLs) of Yb^3+^ and Tm^3+^ was exploited. Even at extreme temperatures, these NIR bands are quite intense, and their intensity ratio fluctuates dramatically, allowing accurate temperature detection. The sensitivities of these two methods are 2.86% K^−1^ (at 300 K) and 2.13% K^−1^ (at 1009 K), as shown in Figure 3.

Recent results of the typical nanothermometry explorations of lanthanide-based upconverting nanoparticles are summarized in Table 2.

Despite the fact that there have been few reports on the use of divalent lanthanide ion-activated phosphors in luminescence thermometry, we are not aware of the usage of corresponding nanoparticles.

## 3. Transition Metal Ion-Activated Nanoparticles

**Figure 4:**
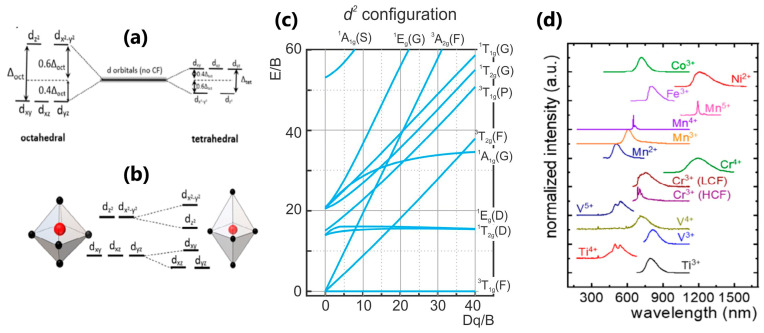
(**a**) TM ion’s energy levels splitting in octahedral and tetrahedral environment; (**b**) the influence of the polyhedron distortion on *3d* energy levels splitting; (**c**) Tanabe–Sugano diagram for the *d^2^* configuration; (**d**) representative emission spectra of different TM ions. Adapted from [10] under the Creative Commons Attribution License.

Transition metal ion-activated photoluminescent nanoparticles are a broad range of compounds that, together with lanthanide-doped nanoparticles, are commonly employed in luminescent thermometry. In this context, ions of transition elements belonging to the fourth period of the periodic table (namely, from ^22^Ti to ^30^Zn) function as optically active centers, which are included in insulating materials or large band gap semiconductors. When a transition metal ion (TM) is incorporated into a solid host, its outer *4s* electrons are removed, leaving partially filled *3d* shells as optically active [49]. Therefore, the electronic configuration of TM ions is *3d^n^* (0<n<10), where partially occupied *d* orbitals provide various energy levels for possible optical transitions. Correspondingly, all TM ions with the same configuration exhibit similar optical properties [4]. In contrast to the *4f*-orbitals of lanthanide ions, *d*-orbitals are not protected from the outside environment, making TM ions highly sensitive to the features of the materials host, particularly its crystal field strength [49]. All this implies that the spectroscopic properties of TMs as optical probes are the consequence of the specific ion’s electronic configuration, as well as the crystal field potential of ligands [4]. The influence of a host crystal lattice on a TM ion inside it is elucidated using crystal field theory [50], where the TM ions are subjected to the electrostatic field from the surrounding atoms (ligands) and treated as negative point charges located in the corners of the coordination polyhedron. Thus, the crystal field of the ligands causes perturbation of the energy levels of *d*-orbitals, which is usually much stronger (~10^4^ cm^−1^) than the spin-orbit interaction (~10^2^ cm^−1^). The total separation of *3d* energy levels—the *crystal field strength* (CFS)—is denoted by *Δ* or *10Dq*, and it strongly depends on the coordination number and symmetry of the crystalline environment. In Figure 4a, the splitting of the *3d* energy levels in octahedral and tetrahedral environments is presented [10]. Further separation can occur via the so called Jahn–Teller effect, with the deformation in the coordination polyhedron (Figure 4b) [51]. Other parameters of interest in the crystal field theory are the so called Racah parameters *A*, *B*, and *C*, where *B* is the most important one and represents the individual *d* electron repulsion [4]. The important and useful tools for the prediction and explication of the TM-doped phosphors’ spectroscopic properties are the Tanabe–Sugano diagrams, calculated for each electronic configuration in an octahedral crystal field [52]. They provide the energy layout of the electronic states, with their symmetry and term notations, with respect to the crystal field parameters *Dq/B*. The exemplary Tanabe–Sugano diagram for the *d^2^* configuration (exists in Ti^2+^, V^3+^, Cr^4+^, Mn^5+^, Fe^6+^ ions) is shown in Figure 4c. The free ion energy levels correspond to Dq/B=0. The lowest energy level, the ground state, coincides with the *x*-axis. The free ion levels are noted as L2S+1, where the values for L can be 0 (indicated by *S*), 1 (*P*), 2 (*D*), 3 (*F*), 4 (G). When incorporated into the crystal field, a TM ion’s levels are given by X2S+1, where X can be *A* (no degeneracy), *E* (double degeneracy, doublet), or *T* (triplet) [49]. The increase in the crystal field strength does not influence all the levels in the same way—the levels with the higher slope in the diagram are more sensitive to the Dq/B values. Figure 4d displays representative transition metal ions that are most frequently used in luminescent thermometry, and their emissions span from the UV to NIR spectral range [10].

The Cr^3+^ ion was one of the first transition metal ions to be used in luminescence temperature measurements [53,54]. It is also the most widely employed of all the TM ions, followed by manganese ions of different valence (Mn^2+^, Mn^3+^, Mn^4+^, Mn^5+^), while other ions of interest are Ti^3+^, Ti^4+^, V^3+^, V^4+^, Fe^3+^, Co^2+^, and Ni^2+^ [10]. In the ever-growing field of biological research, ions like Ti^2+^, Co^2+^, Ni^2+^, V^2+^, and Cr^4+^ are finding their use in temperature measurements of tissues and cells in all three biological windows [4]. When discussing transition metal ion-doped nanoparticles, various temperature-dependent spectroscopic properties can be monitored, such as changes in TM ion emission intensities, emission bandwidths, bandshifts, or excited state lifetimes, as well as the intensity ratios between different emission bands. In order to explain and quantify these changes, different theoretical models have been proposed [7,10]. Generally speaking, in this type of luminescent nanoparticle, both radiative and non-radiative transition rates alter with temperature, making their luminescence intensity highly sensitive to temperature fluctuations, which results in excellent thermometric performances [55,56,57]. The advantages of using TM ions as optical probes over lanthanide ions, which have undoubtedly dominated this research area, lie in their distinctive spectroscopic properties. As they strongly depend on the type of material into which they are placed, their temperature-changing characteristics can be modulated by choosing the appropriate matrices [58]. Furthermore, they have much higher absorption cross-sections than that of the *f–f* transitions of Ln^3+^ ions, allowing for enhanced luminescence brightness. Very often, hosts are being co-doped with lanthanide and transition metal ions, which may offer interesting phenomena and improved thermometric properties [57,59,60,61,62]. Lanthanide ions can activate additional methods of thermal quenching of the TM’s excited states, as well as introduce supplementary transitions for ratiometric thermometry methods.

The initial record of using temperature-dependent luminescence behavior in materials doped with transition metal (TM) ions for thermometry was reported in 1997 by Fernicola et al. [53]. In their study, they developed fiber optic thermometers using Cr-doped forsterite and olivine crystals. The researchers investigated the decay of strong near-infrared emission throughout the temperature range of 77–373 K. They discovered a significant decrease in the emission lifetime, which was shown to be four times shorter across this temperature range. Nevertheless, the present analysis lacks information regarding this sensitivity.

**Figure 5:**
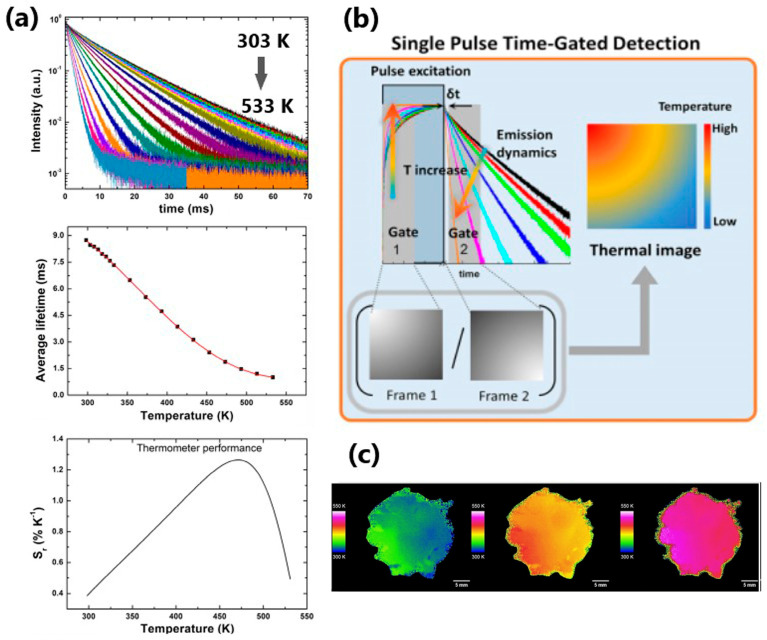
(**a**) Temperature evolution of the emission decay, average lifetime, estimated relative thermal sensitivity; (**b**) schematic representation of the real-time thermal imaging method using single pulse time-gated detection. Frames 1 and 2 are acquired during and after the laser excitation pulse. The thermal image is obtained by the ratio of frames 1 and 2 for every excitation pulse; (**c**) thermal image obtained using single pulse time-gated method. Adapted from [63] under the Creative Commons Attribution License.

An interesting example of the Cr^3+^ (*d^3^* configuration) use in nanothermometry is presented in the work of Avram et al. [63]. The authors synthesized Cr^3+^-doped zinc gallate (ZnGa_2_O_4_:Cr) nanoparticles and co-doped them with Ge^4+^, obtaining the particle size of ~50 to 80 nm. The characteristic spin-forbidden ^2^E → ^4^A_2_ transition of the Cr^3+^ ion that appears around 700 nm in the emission spectrum was monitored. The lifetime of this transition is in the milliseconds range, and it was used for luminescence thermometry in the temperature span of 303–533 K. The ZnGa_2_O_4_:0.05Cr nanoparticles exhibited the best performance, with the maximum sensitivity of 1.25% K^−1^ at 493 K (Figure 5a). These authors have also developed an experimental setup for real-time thermal imaging (the method is schematically presented in Figure 5b and the thermal image obtained by it in Figure 5c).

**Figure 6:**
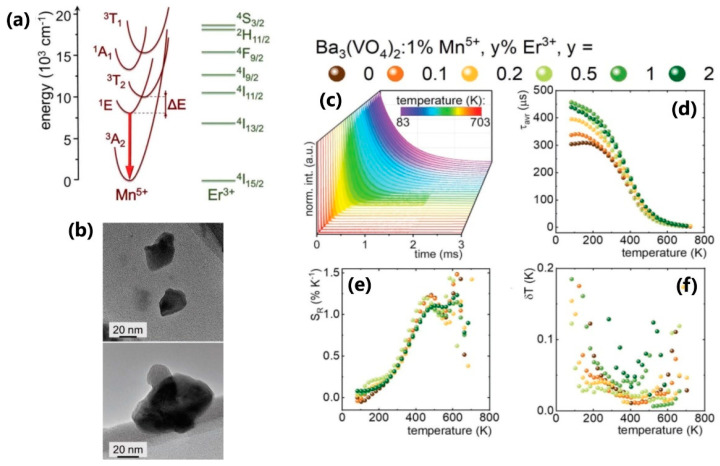
(**a**) Configurational coordinate diagram for Mn^5+^ (*d*^2^ configuration, in the tetrahedral environment, the Tanabe–Sugano diagram for *d*^8^ configuration is applied) and Er^3+^ ions; (**b**) the representative TEM images of Ba_3_(VO_4_)_2_:1%Mn^5+^,0.5%Er^3+^ nanoparticles; (**c**) thermal evolution of luminescent decays of ^1^E excited state of Mn^5+^ ions for Ba_3_(VO_4_)_2_:1% Mn^5+^, 0.5% Er^3+^; (**d**) thermal evolution of Mn^5+^ average lifetimes; (**e**) the relative sensitivities; (**f**) temperature uncertainties for Ba_3_(VO_4_)_2_:1%Mn^5+^ co-doped with different Er^3+^ concentrations. Adapted from [64] under the Creative Commons Attribution License.

The Mn^5+^ ion has recently been proven as an important luminescent activator for biological applications. Its intense and narrow emission band lies in the second biological window, while the excitation is in the first biological window. There are certain prerequisites for these features— a tetrahedral crystal environment, a sufficient host’s energy band gap, as well as the suitable composition that enables the stability of the 5+ valence state [10]. A very recent report on accurate deep-tissue thermal monitoring using a Mn^5+^ ion was published by Piotrowski et al. [64]. The authors examined the temperature dependence of the excited level lifetimes of Mn^5+^-doped Ba_3_(VO_4_)_2_ nanoparticles of the average size of ~52 nm, as well as the influence of co-doping with different lanthanide ions (Nd^3+^, Pr^3+^, Tm^3+^, Er^3+^), with the main results shown in Figure 6. The emission transition ^1^E → ^3^A_2_, which appears at 1178 nm and reaches lifetime values of 450 µs, served for thermal probing. The optimized composition of the nanothermometer is Ba_3_(VO_4_)_2_:1%Mn^5+^,0.5%Er^3+^, which features relative thermal sensitivity between 0.5 and 1.2%K^−1^ in the 350–500 K temperature range. This research also contains the proof-of-concept experiment of thermal imaging in vivo.

In Table 3, the most recent results of luminescence thermometry studies using the nanoparticles activated by transition metal ions are shown.

At the transition from bulk to nanocrystalline lanthanide and transition-activated materials, certain thermometric properties are maintained (ratiometric), while others can significantly differ (emission decay times and emission bandwidths). It is therefore useful to look at these properties in bulk materials, which are given, for example, in reference [4].

## 4. Semiconductor Quantum Dots

**Figure 7:**
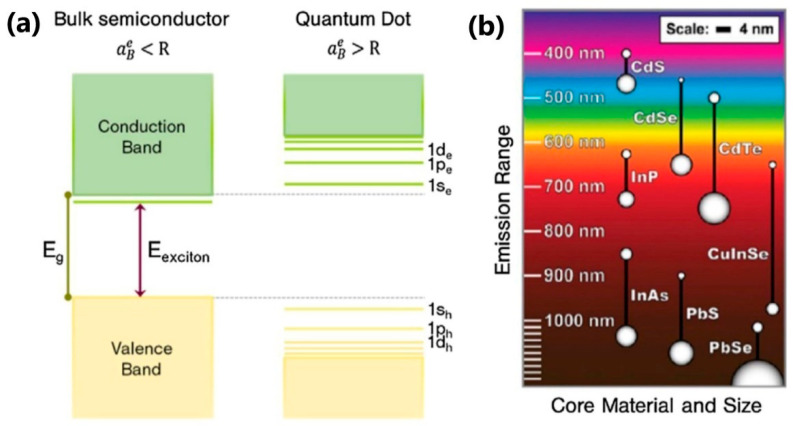
(**a**) Schematic presentation of the discretization of the energy levels in QDs, relative to the bulk material; (**b**) luminescent shifts of different QDs of different sizes. Reprinted with permission from [14].

Semiconductor quantum dots (QDs) are defined as semiconductor nanocrystals, i.e., inorganic particles in the size range of 1–10 nm, that are generally composed of II-VI and III-V elements [75,76,77,78]. Currently, QDs are also based on I–VI, IV–VI, I−III−VI elements, as well as transition-metal dichalcogenides, perovskites, and carbon [76,79]. Their zero-dimensionality causes the quantum confinement of their charge carriers and some unique and fascinating optical properties of QDs arise—sharp and symmetrical emission spectra, high quantum yield, good chemical and photo-stability, and size-dependent emission wavelength tunability [80]. In addition, they display acceptable biocompatibility and biofunctionality after capsulating and/or surface modification [18,79]. The size of QDs can be controlled by regulating nanometer accuracy during chemical synthesis, which results in the adjustability of their emissions from the UV to the near-infrared spectral range. Like all semiconductors, the QDs possess the valence- and the conduction band, separated by a band gap. Their charge carriers are formed when the electrons are excited to the conduction band, leaving holes in the valence band. The electron-hole pairs, bounded through the Coulomb interaction, are called excitons. As the size of the QD reduces, quantum confinement in both the electron and hole wavefunctions leads to an increase in the QD’s effective bandgap and the appearance of the discrete energy levels in the vicinity of the valence- and conduction band. Hence, different optical transitions become possible and luminescence can be tuned [14,18]. The critical parameters that determine QDs’ luminescent properties are as follows: QD’s particle radius (*R*) and Bohr radius (electron-hole distance in an exciton, $a_{B}^{e}$). The mutual relationship of these quantities, as well as the influence of *R* on luminescent color in various QDs, are illustrated in Figure 7.

From the point of view of thermal sensing, several properties of the quantum dots’ emission are affected by temperature—intensity, lifetime, peak position, and Stokes-shift (spectral separation between absorption and emission). The simplest of them is the emission intensity thermal evolution, but it is influenced by the particle concentration and excitation power fluctuations [14]. The method based on the temperature-dependent spectral shift of the emission wavelength is the best known [81], and it overcomes the drawbacks of the intensity-based methods, as the thermal reading is unaffected by the local variations [78]. This phenomenon occurs as a consequence of different effects, such as bandgap energy thermal change, quantum effects, electron phonon coupling, quantum yield variations, and the thermal expansion of the crystalline lattice [81]. The temperature dependence of QD luminescence is a complex phenomenon in which both the nature and the magnitude of the thermally originated changes depend on the QDs’ size [4]. Introducing impurities to semiconducting quantum dots (mostly Mn^2+^ and Eu^3+^) may offer additional possibilities in terms of temperature read-outs, as these bring in new levels inside or outside the QDs’ band gaps [4,82,83].

To the best of our knowledge, the luminescence temperature dependence of QD nanoparticles was originally observed by Joly et al. in 2001. They explored the luminescence behavior of ZnS:Mn^2+^ at temperatures 11–273 K and discussed its mechanisms [84]. A bit later, Wang et al. reported the thermal behavior of CdTe nanoparticles (~4 nm size, 30–60 °C temperature range, 350 nm excitation), and found the emission intensity decreased linearly and reversibly with a sensitivity of 1.1%/°C [82]. These results paved the way for future investigations in this field. One of the first demonstrations of suitability of using QDs for temperature sensing in vitro was demonstrated in 2010 by Maestro et al. [85], who examined CdSe dispersed in phosphate buffered saline for biomedical imaging by measuring the temperature evolution of a single HeLa cancer cell.

**Figure 8:**
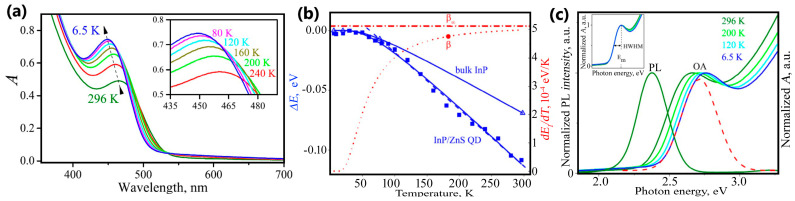
(**a**) Optical absorption spectra of InP/ZnS at various and temperatures; (**b**) energy shift and broadening analysis of exciton absorption band: ΔE1 temperature dependence (blue square symbols are experimental estimates); (**c**) normalized PL and OA spectra of the InP/ZnS QDs. Adapted from [86].

The exciton absorption band shift can also be used for temperature measurement, as shown by Savchenko et al. [86]. These authors have tested the optical absorption spectra ranging from 6.5 to 296 K of colloidal InP/ZnS core-shell quantum dots with the coating of a modified polyacrylic acid. The results show that the first exciton absorption band, which is attributed to the InP exciton band, shifts towards higher energies when cooled from room temperature. The obtained experimental data have been approximated by means of a linear model and Fan’s expression (presented in Figure 8).

**Figure 9:**
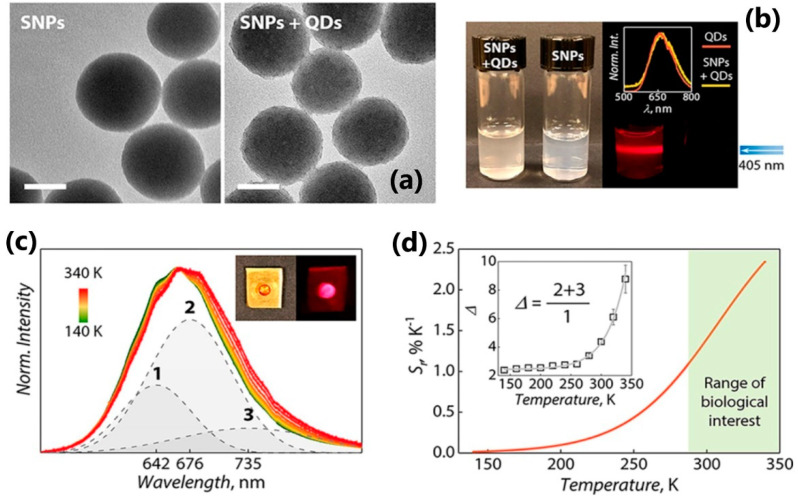
(**a**) Silica nanoparticles (SNPs), before and after decoration with MPTS-passivated CIS QDs (50 nm scale bars); (**b**) suspensions of SNPs and SNPs decorated with QDs under daylight (left) and 405 nm excitation (right, image taken with a 500 nm long pass filter). In the inset, the normalized spectra of a QD sol in chloroform and the SNPs decorated with QDs suspended in water are presented; (**c**) temperature-dependent emission of a polymeric film embedding MPTS-passivated CIS QDs under 405 nm excitation (solid lines) along with exemplary Gaussian curves used for the deconvolution of the signal recorded at 250 K. In the inset, the polymeric film under daylight and UV light is shown; (**d**) relative thermal sensitivity of the thermometric approach and corresponding thermal parameter (Δ, inset) over the investigated temperature range. Reprinted with permission from {MARIN, R.; VIVIAN, A.; SKRIPKA, A.; MIGLIORI, A.; MORANDI, V.; ENRICHI, F.; VETRONE, F.; CERONI, P.; APRILE, C.; CANTON, P. MERCAPTOSILANE-PASSIVATED CuInS_2_ QUANTUM DOTS FOR LUMINESCENCE THERMOMETRY AND LUMINESCENT LABELS. ACS APPL. NANO MATER. 2019, 2, 2426−2436}. Copyright {2023} American Chemical Society [87].

Recently, Marin et al. have produced two types of nanocomposite materials based on mercaptosilane-passivated CuInS_2_/ZnS core-shell quantum dots, the performance of which was tested for luminescence thermometry and luminescent labels [87]. These QDs were deposited onto silica nanoparticles and embedded into polymeric film. Luminescence thermometry was performed on the nanocomposite QD-based film in the temperature range of 140–340 K. The authors monitored the thermal parameter (*Δ*) as the ratio between the sum of integrated area of the two red-most emission components (2) + (3), and the one centered at 642 nm (1) (Figure 9c). The obtained thermal sensitivity reached values of ~2.3%/K.

Recent results of the typical nanothermometry explorations of quantum dots are summarized in Table 4.

## 5. Polymer Nanoparticles

**Figure 10:**
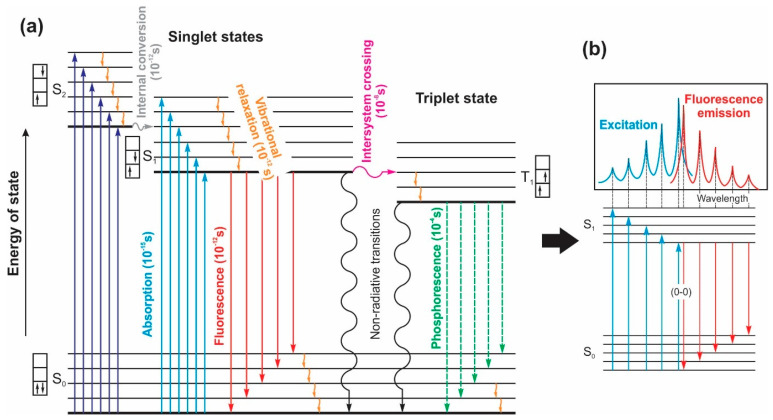
(**a**) Jablonski diagram of the energy of states in organic molecules; (**b**) scheme of the typical spectra of organic molecules.

Organic substances demonstrating photoluminescent properties can be categorized into various classes, including organic dyes, pigments, polymers, and proteins [14,18,78]. The fundamental basis for luminescence in organic compounds is in the π-electron systems present within individual molecules. These systems give rise to electronic transitions between molecular orbitals, leading to the absorption and emission of light [94]. The elucidation of luminescence in organic molecules can be effectively explicated by the renowned Jablonski diagram (Figure 10a). Starting with the initial state known as the ground state, typically denoted as S_0_ and characterized by singlet spin, the molecule undergoes a transition to higher energy states, namely singlet (S_i_) or triplet (T_i_) states, upon the absorption of a photon. These excited states, with i being greater than or equal to 1, exhibit distinct vibrational and rotational energy levels. Then, the molecule quickly relaxes from the higher energy excited singlet states (in the ps range) to the lowest singlet state *S_1_* through *internal conversion* (*IC*), from where it decays back to the ground state *S_0_* either radiatively (*fluorescence*) or nonradiatively (through IC or *external conversion, EC*). Alternatively, the molecule can undergo an *intersystem crossing* (*ISC*) (in the 10 ns range) and transit to a triplet state. It should be noted that the singlet –singlet transition, $S_{1}\to S_{0}$, is more probable than the singlet–triplet transition, $S_{1}\to T_{1}$, as the latter is spin-forbidden since it involves a spin multiplicity change. From there, the $T_{1}\to S_{0}$ transition can be either radiative (*phosphorescence*) or nonradiative (ISC or EC). The time frame of the phosphorescence can even be in the millisecond range, precisely because of its forbidden nature [94]. The spectral properties of organic compounds arise from these transition mechanisms, and often contain a specific vibronic structure as well, as schematically illustrated in Figure 10b.

The flexibility of organic compounds is considered to be one of their primary advantages in comparison with other thermometric nanoparticles. A diverse array of organic fluorophores is already accessible, each engineered to possess distinct characteristics such as absorption and emission wavelengths, spectrum range, solubility, and functionalization capabilities [18]. Furthermore, these temperature sensors have the capability to be seamlessly integrated with other organic or inorganic species, resulting in the formation of hybrid sensors [4]. Although the temperature-dependent quenching mechanism may differ between compounds, the generalized, fundamental photodynamics can be explained using the Jablonski energy-level model. The relative probability for the excited molecule to descend to the ground state via the radiative or nonradiative path(s) changes when the temperature changes, effectively modifying the molecule’s luminescence intensity (and lifetime). The variation in decay rates with temperature is a consequence of complex and sometimes competing effects [18]. The most utilized thermometric organic dyes are fluorescent, such as fluorescein of rhodamine B, and their emission and lifetime change with temperature [14]. Green Fluorescent Protein (GFP) is an interesting example of in vivo temperature measurements and fluorescence imaging at the cellular level, because its temperature-dependent characteristic is fluorescence polarization anisotropy [78]. One should also mention the application in aerodynamics, where organic dyes are mixed with paints to form pressure-sensitive paints (PSP), which are used to monitor pressure and temperature on aircraft model surfaces [18]. Qiao et al. [95] classify the fluorescent polymeric nano-thermometers into two categories: thermosensitive polymer-based and nonthermosensitive polymer-based. Both groups comprise a polymer and organic fluorescent dye. Thermosensitive polymer undergoes a reversible phase transition with temperature that induces a change in the optical properties of a dye incorporated in it. Nonthermosensitive polymer serves only as a matrix of physically embedded optically active organic dye [95].

**Figure 11:**
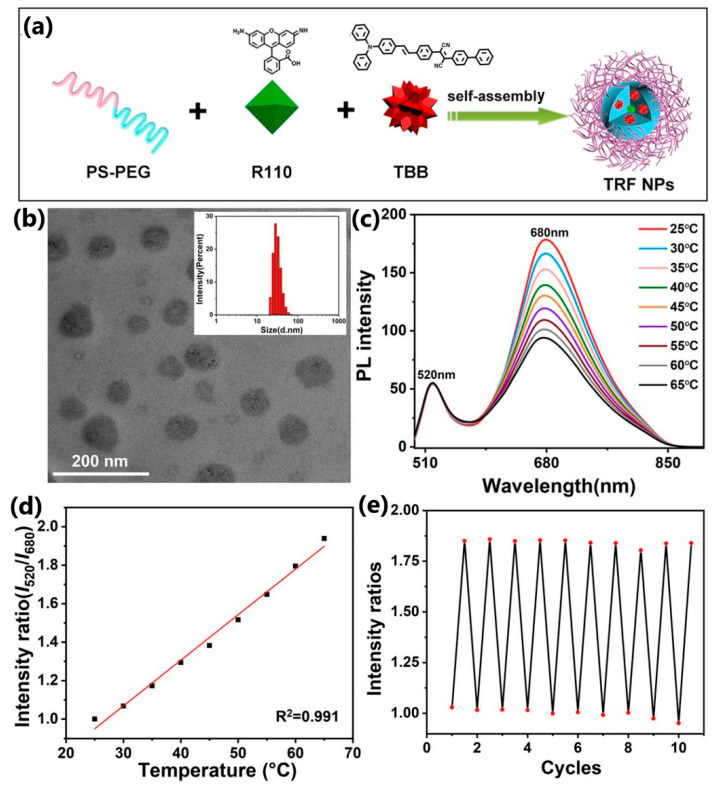
(**a**) Schematic of the fabrication of TRF NPs; (**b**) TEM photographs of TRF NPs, inset: particle size distribution of TRF NPs (mean diameter of 48 nm); (**c**) PL spectra of TRF NPs with temperature increased from 25 to 65 °C; (**d**) PL intensity ratio versus temperature curves of TRF NPs; (**e**) PL intensity ratios variation in TRF NPs during repeated heating and cooling. Reprinted with permission from {MENG, L.; JIANG, S.; SONG, M.; YAN, F.; ZHANG, W.; XU, B.; TIAN, W. TICT-BASED NEAR-INFRARED RATIOMETRIC ORGANIC FLUORESCENT THERMOMETER FOR INTRACELLULAR TEMPERATURE SENSING. ACS APPL. MATER. INTERFACES 2020, 12(24), 26842–26851}. Copyright {2023} American Chemical Society [96].

Meng et al. [96] have monitored the intracellular temperature of Hep-G2 cells under photothermal therapy using the ratiometric fluorescent thermometer. Thermometric organic nanoparticles were prepared by encapsulating thermoresponsive NIR fluorophores (dyes)—TBB (2-([1,1′-biphenyl]-4-yl)-3-(4-((E)-4-(diphenylamino)styryl) phenyl) fumaronitrile) and Rhodamine 110—into an amphiphilic polymer matrix F127 to form TBB&R110@F127 nanoparticles of about 50 nm size and a narrow size distribution (Figure 11b). Upon the 480 nm excitation, TRF NPs exhibited double peaks (520, 680 nm) originated from two different fluorophores. The ratio of these peaks was used to measure thermographic properties in the temperature range of 25–65 °C, yielding a relative sensitivity of 2.37%·°K^−1^ (Figure 11b,c). Also, the stability of this polymer thermometer was confirmed by repeating cycles of heating and cooling [96].

**Figure 12:**
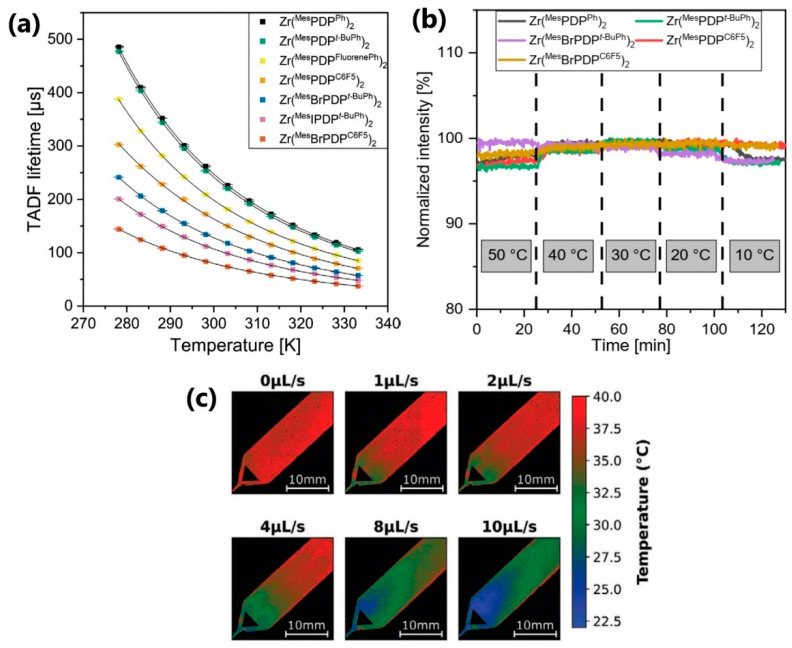
(**a**) Temperature dependency of TADF decay times for Zr(PDP)_2_ complexes immobilized in PS acquired under anoxic conditions; (**b**) Temperature dependence of emission intensity of Zr(PDP)2 complexes immobilized in PS measured under anoxic conditions; (**c**) Imaging of temperature distribution in a microfluidic chip with an aqueous dispersion of Zr(MesIPDP*t*-BuPh)2-PVA-MAA nanoparticles. Adapted from [97] under the Creative Commons Attribution License.

Another example of organic thermographic nanoparticles was recently proposed by Russegger et al. [97], and the results are presented in Figure 12. The authors synthesized different organic zirconium(IV)-pyridinedipyrrolide complexes as dyes and immobilized them in gas-blocking polymers. In this way, they obtained negatively charged PVA-MMA-based nanoparticles for temperature imaging in microfluidic devices, and positively charged PVA-TMA-based nanoparticles for thermometry in live cells. The size of PVA-MAA particles was ~34 nm with a uniform size distribution, while for PVA-TMA particles the size distribution was trimodal (~9 nm, ~42 nm and ~344 nm). The temperature-dependent property of interest was a luminescence decay time of different Zr-complexes in the temperature range 5–60 °C, while the emission intensity remained unchanged (excitation at 530 nm) (Figure 12a,b, respectively). Mono-exponential decay in the order of tens and hundreds of microseconds exhibited temperature sensitivities between −2.5 and −2.9% K^−1^ in polystyrene at 25 °C [97].

Recent results of the typical nanothermometry explorations of polymer nanoparticles are summarized in Table 5.

## 6. Carbon Dots

**Figure 13:**
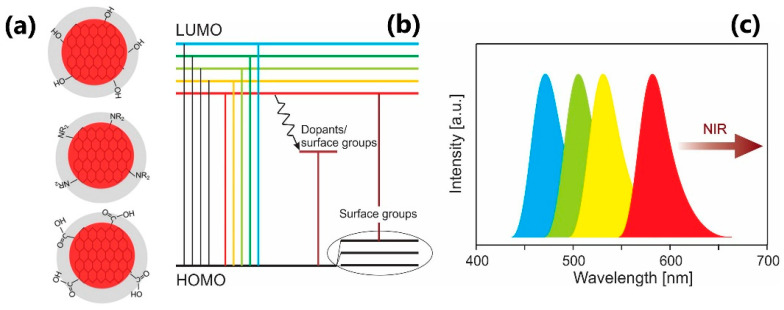
(**a**) Schematic presentation of the CDs with the exemplary functional groups at their surface. Red color indicates a graphene-like core and the grey color stands for the surface functional groups surrounding the core. Adapted with permission from [115]; (**b**) simplified energy band scheme of CDs. Dopants and/or surface functional groups can introduce additional energy levels and modify possible photoluminescence output; (**c**) typical luminescence emission spectra of various CDs can appear in different spectral ranges, from blue to NIR.

Carbon dots (CDs) are zero-dimensional carbon-based materials that have a size range of a few tens of nanometers [107]. They can be chemically modified and/or doped to improve or add useful qualities. CDs have extraordinary features, including tunable optoelectronic capabilities, bright photoluminescence with high quantum yields, non-toxicity, and superior biocompatibility, as well as modifiable functional groups that come along with simple and cost-effective manufacturing procedures [108,109,110,111]. Most of the CDs consist of a sp^2^/sp^3^ carbon skeleton with possible functional groups or polymer chains. Their core can have a graphite/diamond lattice or carbon in amorphous form, depending on the different degrees of carbonization [111]. The origin of luminescence in carbon dots is still a subject of ongoing investigation and debate. CDs manufactured using various techniques, precursors, and post-treatments have distinctive optical properties, indicating that CDs present a sophisticated system. There are several proposed mechanisms for the luminescence of carbon dots, and it is likely that multiple factors influence it [109]:

- *Surface States and Functional Groups*: Carbon dots have a high density of surface states—the electronic states are localized near their surface. These surface states can arise from the presence of oxygen and functional groups such as carboxyl, hydroxyl, or amino groups on the surface of the carbon dots, schematically shown in Figure 13a. The interaction between these surface states and the excited electrons can lead to radiative recombination, resulting in the emission of light [112], Figure 13b,c.
- *Quantum Confinement Effect*: The small size of carbon dots leads to quantum confinement effects, which can influence the electronic structure of the carbon material, causing the formation of discrete energy levels [113].
- *Molecular Fluorescence*: Introducing fluorescent impurities (organic molecules, in particular) during the synthesis may contribute greatly to the emission of CDs [114], Figure 13b,c.

The mechanism of carbon dots’ temperature-dependent luminescence is still not clearly explained. Typically, with an increase in temperature, the exitonic emissions from CDs are redshifted and nonradiative decay rates increase [116]. Yang et al. [117] ascribe thermal linear fluorescence quenching to the synergistic effects of plentiful oxygen-containing functional groups and hydrogen bonds. On the contrary, other authors attribute linear fluorescence temperature decay to the temperature-induced “energy traps” on the CDs’ surface, the non-radiative channels of trap/defect states that become activated at higher temperatures, thus provoking energy transmission and thermal quenching [118,119,120]. Yu et al. [118] were the first authors to investigate the temperature-dependent fluorescence in carbon nanodots by measuring their temperature-dependent photoluminescence lifetimes within the 77–300 K range and comparing them to semiconductors and metal-based nanoparticles.

**Figure 14:**
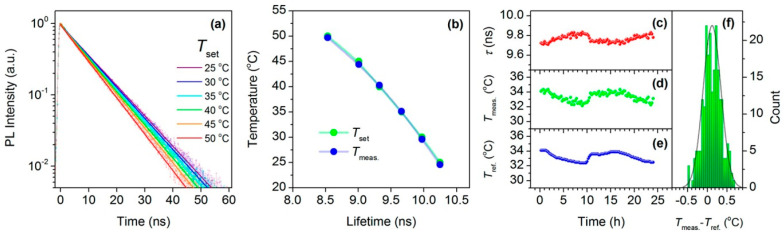
In vitro intracellular PL lifetime thermal sensing using N,S-CDs. (**a**) PL emission decays of HeLa cells incubated with N,S-CDs (500 μg/mL) at different temperatures (T_set_), together with the single-exponential fits; (**b**) temperatures determined using the calibration curve (T_meas._) and set temperatures (T_set_) plotted against the PL lifetime; (**c**–**f**) applicability of N,S-CDs for long-term remote intracellular temperature monitoring; (**c**) PL lifetimes extracted from PL transients recorded every 15 min for 24 h of HeLa cells incubated with N,S-CDs (500 μg/mL); (**d**) temperatures determined using the calibration curve; (**e**) temperatures measured with a reference thermometer (T_ref._); (**f**) histogram showing the distribution of temperature differences between the obtained and reference temperatures; the solid line is the distribution curve. Reprinted from [121] under an ACS Author Choice License.

Kalytchuk et al. [121] obtained highly luminescent water-soluble nitrogen and sulfur-codoped CDs and examined their photoluminescent properties in a wide range of temperatures (10–70 °C). In contrast to many semiconducting nanocrystals, the absorption and emission spectra of N,S-codoped CDs did not show any band shifts at different temperatures. On the other hand, they exhibited temperature-dependent emission decays and can serve as highly sensitive intracellular nanothermometers, stable over a wide range of pH values, CD concentrations, and environmental ionic strengths, with a maximum sensitivity of 1.79%K^−1^ and a statistical accuracy of 0.27 °C. The results enabled the authors to achieve in vitro photoluminescence lifetime-based temperature sensing in human cervical cancer (HeLa) cells (shown in Figure 14). Moreover, the absolute PL quantum yield of these CDs is as high as 78 ± 2% under a 355nm excitation.

**Figure 15:**
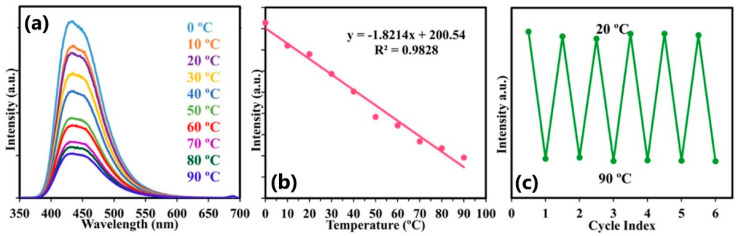
(**a**) Fluorescence spectra of CDs at different temperatures; (**b**) the linear fit of fluorescence intensity temperature change; (**c**) fluorescence intensity upon the cyclic switching of CDs under alternating conditions of 20 °C and 90 °C. Reprinted from [122], under the Creative Commons Attribution 4.0 International License http://creativecommons.org/licenses/by/4.0/, accessed on 11 October 2023.

Mohammed et al. [122] showed that their N,B-CDs’ photoluminescent emission spectra are very sensitive to temperature changes within the 0–90 °C range, as presented in Figure 15. These CDs emitted blue fluorescence that peaked at 450 nm and exhibited up to 70% quantum yield, which can be used for highly sensitive temperature measurements with a thermo-sensitivity of 1.8%°K^−1^, excellent recovery, and pH stability. Apart from being excellent nanothermometers, these CDs can be used as Fe^3+^/Fe^2+^ sensors in biological samples.

In the context of detecting temperature fluctuations in the surrounding environment, the utilization of ratiometric optical nanothermometry is advantageous due to its reliance on the ratio between two luminescent signals. This approach offers benefits such as self-calibration of the system and enhanced reliability in thermal measurements [115,116].

Recent results of the typical nanothermometry explorations of carbon dots are summarized in Table 6.

## 7. Nanodiamonds

The research regarding nanodiamonds (i.e., diamond nanoparticles) was initiated in the Soviet Union during the 1960s [131]. Nanodiamonds combine many superior properties of bulk diamond. Some of the most important characteristics are summarized in [132], such as chemical inertness, wide-band gap electronic properties, excellent thermal conductivity, and outstanding mechanical behavior to those conferred by their high specific surface area [131]. In addition, they are non-toxic and have rich surface chemistry, making them ideal candidates for biomedical in vivo and in vitro applications [133,134,135]. The luminescent properties of nanodiamonds arise from the defects within their crystal lattice that introduce energy levels within the bandgap of the diamond structure, causing absorption and, subsequently, the emission of light. The negatively charged nitrogen-vacancy color center (NV^−^, or just NV) is one of the 500 distinct color centers discovered in diamond, and one of the most promising so far [136]. Silicon-, germanium-, tin-, and lead-vacancy color centers (SiV, GeV, SnV, and PbV, respectively) are also of interest [137]. The thermometry methods in nanodiamonds are categorized into *spin-based thermometry* and *all-optical thermometry*, based on their temperature-dependent features. The NV centers are employed in both spin-based and all-optical thermometry, whereas the other color centers are predominantly used in all-optical thermometry [136,137].

**Figure 16:**
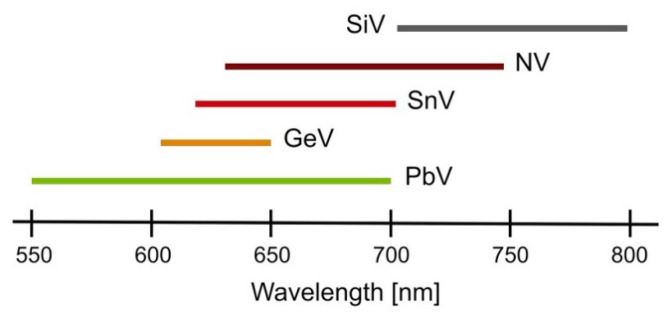
Wide fluorescent spectral range of different color centers in diamond. Reprinted from [137], under the Creative Commons Attribution 4.0 International License http://creativecommons.org/licenses/by/4.0/, accessed on 11 October 2023.

The NV color center comprises a C-atom vacancy and a N-atom impurity plus an additional electron, trapped at the defect center, and has C_3v_ point group symmetry [131,138,139]. The photoluminescence spectra of the NV center lie in the visible range (Figure 16). They can be excited by easily accessible optical lasers (532 nm), whereas the emission spectra of the NV center consist of a Zero-Phonon Line (ZPL) at 638 nm accompanied by broad phonon side-bands (PSB) that originate from thermally excited vibrational states [140]. The peculiarity of the NV center photoluminescence is its dependence on the temperature and magnetic field when subjected to resonant microwave excitation. These features are the foundation of two different thermometry methods in nanodiamond—zero-phonon line (ZPL) thermal shift (i.e., all-optical thermometry) and optically detected magnetic resonance (ODMR) thermal shift (i.e., spin-based thermometry) [131,136,137,140].

**Figure 17:**
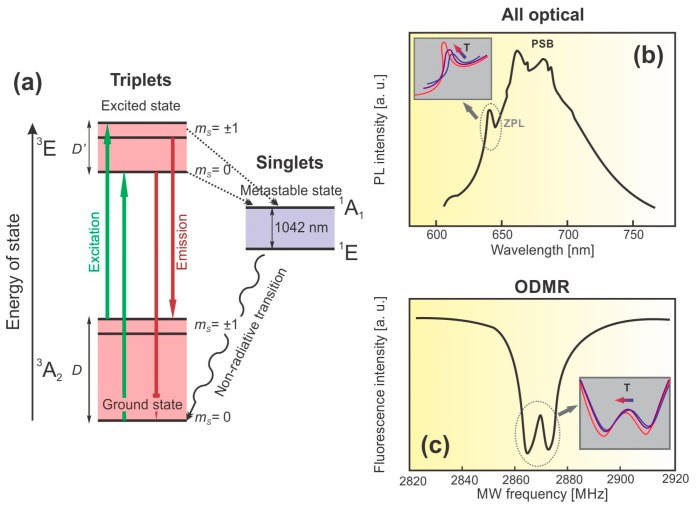
(**a**) Basic energy level scheme of the NV center; (**b**) schematic presentation of the NV center all-optical thermometry, typical photoluminescence emission spectrum of a single NV center displaying ZPL (638 nm) and PSB, in the inset: the temperature-dependent shift of ZPL; (**c**) typical ODMR spectra of the NV centers in nanodiamonds, in the inset: thermally induced changes in the ODMR spectra.

The simplified electronic structure of the NV center is presented in Figure 17a. The centers have triplet ground states (^3^A_2_) with electron spin sublevels of $m_{S}=0$ and $m_{S}=\pm 1$, where the spin–spin interaction is responsible for a zero-field splitting of D=2.88 GHz. At room temperature the spin–orbit coupling results in the ^3^E excited state fine structure with three detectable levels of $m_{S}=0$ and $m_{S}=\pm 1$, separated by a zero-field splitting of $D^{\prime }=1.42\;GHz$. The intersystem crossings can take place from the ^3^E excited state to/from the intermediate ^1^E and ^1^A singlet states, causing weak near-infrared (NIR) emissions [131,138,141]. The associated photoluminescence spectra and schematic presentation of the all-optical thermometry method are schematically summarized in Figure 17b. A strong optical transition that occurs between the ^3^A_2_ ground state and the ^3^E excited state (green arrow) initiates the ZPL at 638 nm (red arrow) at room temperature. The position, amplitude, and width of ZPL changes with temperature according to a Lorentzian function with an exponential background. Furthermore, the ratiometric all-optical thermometry method relies upon the ratio of the counts underneath the ZPL and the total emission spectra, known as the Optical Debye–Waller factor. By applying the microwave resonance to the transition between of $m_{S}=0$ and $m_{S}=\pm 1$ in the ground state, the photoluminescence substantially decreases through a process called the optically detected magnetic resonance (ODMR). This method, presented in Figure 17c, analyses the temperature dependence of D, a phenomenon arising from the thermal lattice expansion and temperature dependence of the electron–phonon interaction [131].

The usage of the temperature-dependent fluorescent properties of the NV centres in nanodiamonds for thermometry in the range of 300–700 K was first proposed by Plakhotnik et al. [142]. The authors introduced ratiometric all-optical thermometry method based on the Debye–Waller factor of the NV fluorescence spectrum and obtained the variability in the fluorescence intensity and lifetime of approximately −0.2%/K and −0.06 ns/K, respectively.

**Figure 18:**
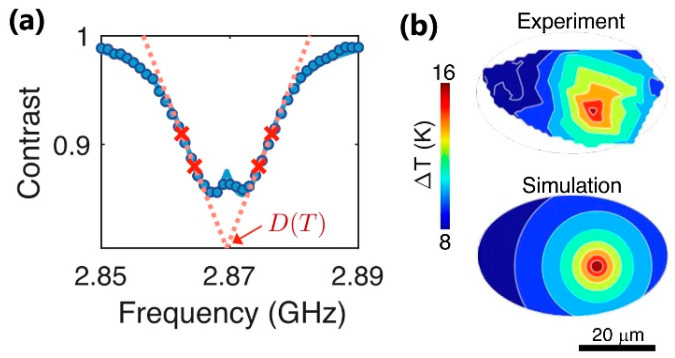
(**a**) Optically detected magnetic resonance of an NV thermometer. Contrast is defined as the fluorescence ratio of the thermometer with and without the application of microwaves. In temperature measurements, the resonance curve is sampled at four optimized frequencies (red crosses) to extract the resonance position D(T) at a given local temperature T; (**b**) 2D temperature distribution map of the laser-illuminated *C. elegans* embryo, measured by a collection of NV thermometers inside an embryo, with comparisons between experiments and simulations. Adapted from [143].

Choi et al. [143] published important results (displayed in Figure 18) about using the nanothermometry method of NV centers in nanodiamonds of ~50 nm size for in vivo temperature measurements as well as for the regulation of cell division timings in *C. elegans* embryos. NV nanothermometers were injected inside *C. elegans* embryos, allowing the in vivo monitoring of the temperature and the correlation of cell-division dynamics with the local temperature distribution inside an embryo. An infrared laser was utilized to achieve heating within the temperature range of ΔT~20 K, commencing from an initial temperature of 12.3 °C. NV temperature sensors were characterized using the ODMR method and had maximal temperature sensitivities of ~2 K/Hz^−1/2^.

**Figure 19:**
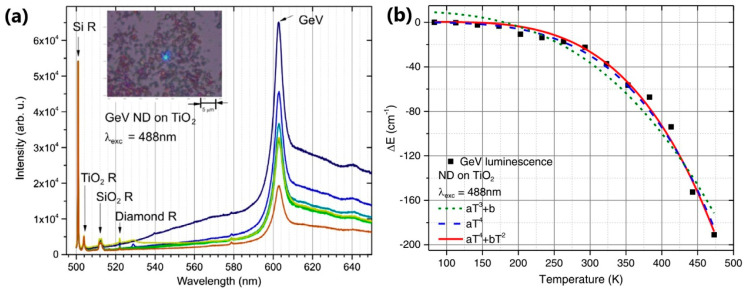
(**a**) Emission spectra of NDs on the surface of TiO_2_ film, upon heating by a focused laser beam. The laser was pointed onto the object for 5 min while recording a spectrum every minute. Then, the laser excitation was removed, and the object was cooled for 5 min, after which, a spectrum was recorded again. The spectra are obtained in the temperature range 293–323 K. Insert: a cluster of ND aggregates; (**b**) the fits of the ZPL shifts from different theoretical models. Adapted with permission from [147].

Unlike NV centers, which exhibit an almost 100 nm broad emission band, group IV-based defects in nanodiamonds concentrate almost all their photoluminescence into a few nanometers-wide zero phonon lines (ZPLs), and their temperature-dependent changes have been used for all-optical thermometry [131,144,145,146,147,148]. Miller et al. [147] studied aggregates of nanodiamonds with germanium-vacancy (GeV) defects distributed on different substrates within a wide temperature range of 85–400 K, with the aim of obtaining 2D optical thermometer with high nanoscale spatial resolution, predominantly for biomedical applications. They observed the thermal shift of the GeV center’s ZPL at 602 nm and fitted it with different models. The results shown in Figure 19 proved that nanodiamond aggregates with GeV center are optical temperature sensors with a sensitivity of 0.2 cm^−1^K^−1^ at room temperature [147].

Recent results of the typical nanothermometry explorations of nanodiamonds with different optically active defects are summarized in Table 7. It is important to note that the sensitivity of thermometry using nanodiamonds is commonly expressed as the uncertainty for a given measurement time. For other types of nanoparticles, sensitivity is defined as the rate of change in a measurement over its uncertainty. Therefore, it is difficult to compare the reported sensitivity values for other types of nanoparticles with those of nanodiamonds.

## 8. Conclusions

Luminescent nanoparticles are widely used as probes in luminescence thermometry. They can be utilized in all temperature read-out methodologies, with both steady-state and time-resolved emission detection and with both downshifting and up-conversion excitations. They can be used in almost all luminescence thermometry applications. The only ones that they cannot be used in are measurements at very high temperatures, because at those temperatures, nanoparticles emit less light and are not as stable as their bulk counterparts. For some applications, they are indispensable. These are primarily biomedical and nanotechnology applications that require high spatial measurement resolutions and/or particle surface modifications. In addition, achieving multifunctional luminescence materials is much easier with nanoparticles. The polyvalent functions of nanoparticles related to thermometry so far are mostly bioimaging, nanoheating, and counterfeiting, but some other functions would not be difficult to envisage. Further, by combining several nanoparticles with different spectral and temperature responses, one can construct a luminescence thermometer to suit any specific demand. To achieve similar goals, it is also possible to use core/(multiple) shell nanoparticles. Considering the number of papers published on luminescence thermometry, lanthanide-activated nanophosphors are the most commonly used probes, with semiconductor quantum dots following them. This is probably due to the large and important field of applications of up-conversion materials in which lanthanide-activated nanoparticles play a crucial role. Regarding semiconductor quantum dots, luminescence thermometry performance differs between nanoparticles of different sizes and morphologies.

## Figures and Tables

**Table 1:** The overview of the most recent literature reports on downshifting lanthanide-based nanoparticles for luminescence temperature sensing.

NPs Size [nm]	Host	Lanthanide Ion	Measurement Method	Temperature Range [K]	Maximal Sensitivity [%K^−1^]	Application	Ref.
50–100	Gd_2_Zr_2_O_7_	Eu^3+^	Ratiometric	300–700	0.92	Temperature sensing	[22]
15–20	TiO_2_	Eu^3+^	Luminescence intensity	293–313	2	Sensing temperature within L929 fibroblast cells	[23]
100–200	LaVO_4_	Eu^3+^	Ratiometric, dual-excited single band, CT band position	98–773	2.3	Remote thermometry	[24]
17 × 25	NaYF_4_	Eu^3+^	Ratiometric, dual-excited single band, resonant to GSA and ESA	123–473	4.11	Remote thermometry	[25]
12 × 25	NaGdF_4_				16.9		
70–80	YVO_4_	Eu^3+^	Ratiometric, dual-excited single band, CT band position	299–466	2.23	Remote thermometry	[26]
		Dy^3+^			2.36		
		Sm^3+^			3.09		
10–30	Gd_2_Ti_2_O_7_	Dy^3+^	Ratiometric, trap emission of host and Dy^3+^ emission band	293–443	0.9	Temperature sensing	[27]
80–100	YAlO_3_	Dy^3+^	Ratiometric, three-level	300–850	0.86	Temperature sensing at high temperatures	[28]
~20	La_2_Zr_2_O_7_	Pr^3+^	Ratiometric	85–705	0.41	Temperature sensing at high temperatures	[29]
~100	YAlO_3_	Nd^3+^	Ratiometric	290–610	1.8	Sub-tissue luminescence imaging in I- and II-biological window	[30]
60–70	YVO_4_	Nd^3+^	Ratiometric	123–398	0.54	Temperature sensing in I- and II-biological window	[31]
~50	CaSrSiO_4_	Tb^3+^	Ratiometric	10–290	0.18	Low-temperature sensor	[32]
~50	Y_3_Al_5_O_12_	Er^3+^, Yb^3+^	Ratiometric (Stark components of Er^3+^)	80–600	1	Temperature sensing in biological windows	[33]
			Ratiometric (emissions of Yb^3+^ and Er^3+^)		0.8		
			Yb^3+^ bandshift and bandwidth change		0.4		
			Yb^3+ 2^F_5/2_ state kinetics		0.86		

**Table 2:** The overview of the most recent literature reports on upconverting lanthanide-based nanoparticles for luminescence temperature sensing.

NPs Size [nm]	Host	Lanthanide Ion	Measurement Method	Temperature Range [K]	Maximal Sensitivity [%K^−1^]	Application	Ref.
~50	NaNbO_3_	Tm^3+^	Ratiometric (1390 nm excitation)	295–363	0.80	Subtissue thermal sensing with deep penetration	[42]
70–150	LaPO_4_	Tm^3+^, Yb^3+^	Ratiometric	293–773	~3	In situ pressure and temperature measurements	[43]
20–50	YPO_4_				~2.3		
15	NaYbF_4_@CaF_2_@ NaYF4:Yb^3+^/Er^3+^@ CaF_2_ core/multishell	Tm^3+^, Er^3+^/Yb^3+^	Ratiometric (Tm^3+^)	10–295	3.06	Ultralow temperature sensing of the contactless luminescence nanoprobe	[15]
			Ratiometric (Er^3+^)		0.82		
10–50	NaYF_4_@ NaGdF_4_:Nd core/shell	Er^3+^/Yb^3+^	Ratiometric (Er^3+^)	283–338	9.3	Temperature monitoring in photothermal therapy	[44]
~25	NaYbF_4_/NaYF_4_:Tm^3+^-Yb^3+^ /NaYF_4_ core/shell/shell	Tm^3+^, Yb^3+^	Ratiometric	303–423	3.9	Temperature detection in bio-systems	[45]
50–150	KLu(WO_4_)_2_	Tm^3+^, Ho^3+^	Ratiometric (Tm^3+^ and Ho^3+^ lines)	300–333	2.84	Estimation of thermal resistance	[46]
40 × 70	YVO_4_	Tm^3+^, Er^3+^/Yb^3+^	Ratiometric (Er^3+^ lines)	267–673	7.4	Temperature sensing	[47]
		Tm^3+^, Ho^3+^, Er^3+^/Yb^3+^	Ratiometric (Er^3+^ lines)		2.4		
20–60	Y_2_O_3_	Nd^3+^/Yb^3+^	Ratiometric (Nd^3+^ and Yb^3+^ lines)	303–333	2.9	Temperature sensing in different temperature ranges	[48]
				423–773	2.3		

**Table 3:** The overview of the most recent literature reports on transition metals-based nanoparticles for luminescence temperature sensing.

NPs Size [nm]	Host	Transition Metal Ion (Co-Doping ion)	Measurement Method	Temperature Range [K]	Maximal Sensitivity [%K^−1^]	Application	Ref.
<100	Y_3_Al_5_O_12_	Ti^4+^/Ti^3+^	Ratiometric, Ti^4+^/Ti^3+^ bands	123–573	0.71	Temperature sensing	[65]
		Ti^4+^/Ln^3+^ (Nd^3+^, Eu^3+^, Dy^3+^)	Ratiometric, Ti^4+^/Nd^3+^ bands		3.70		
30 ± 10	Y_3_Al_5_O_12_	Ti^3+^ (Eu^3+^)	Ratiometric, Eu^3+^/Ti^4+^ bands	123–573	1.37	Temperature sensing	[66]
			Ratiometric, Eu^3+^/Ti^3+^ bands		~0.90		
50 ± 10	Gd_3_Al_5−x_Ga_x_O_12_	Cr^3+^ (Nd^3+^)	Ratiometric, Cr^3+^/Nd^3+^ bands	123–573	1.90	Temperature sensing	[60]
			Ratiometric, Cr^3+^/Cr^3+^ bands		1.30		
70 ± 10	Y_3_Al_5_O_12_	Cr^3+^ (Nd^3+^)	Ratiometric, (Cr^3+^/Cr^3+^ bands, Cr^3+^/Nd^3+^ bands)	123–573	2.64	Temperature sensing	[62]
	Y_3_Ga_5_O_12_				~2		
	Y_3_Al_2_Ga_3_O_12_				2.16		
40–50	ZnGa_2_O_4_/ZnGaGeO_4_/	Cr^3+^	Ratiometric, Cr^3+^/Cr^3+^ bands	295–328	~1	Bioimaging and biosensing	[67]
10–30	La_2_LuGa_5_O_12_	Cr^3+^ (Nd^3+^)	Ratiometric, (Nd^3+^/Cr^3+^ bands)	123–573	1.47	Temperature sensing	[61]
35–55	LaScO_3_	Cr^3+^ (Yb^3+^)	Lifetime	123–573	1.70	Temperature sensing	[68]
~100	Mg_2_SiO_4_	Cr^3+^	Emission intensity	10–350	0.7	Temperature sensing	[69]
			Lifetime		0.8		
			Emission band position		0.85		
22–47.6	Y_3_Al_5_O_12_	V^3+^,V^5+^ (Eu^3+^, Dy^3+^, Nd^3+^)	Ratiometric, (V^3+^/V^5+^ bands)	123–573	4–6	Temperature sensing	[61]
			Ratiometric, (V^3+^/Eu^3+^ bands)		0.4		
			Ratiometric, (V^3+^/Dy^3+^ bands)		3.5		
			Ratiometric, (V^3+^/Nd^3+^ bands)		0.91		
<100	Y_3_Al_5−x_Ga_x_O_12_	V^3+^	Emission intensity	123–573	1.08	Temperature sensing	[70]
		V^4+^			1.34		
		V^5+^			2		
		V^5+^/V^4+^	Ratiometric (V^5+^/V^4+^ bands)		2.64		
<100	GAG, GGG, LuAG, YAP, YAG	Mn^3+^ Mn^4+^	Lifetime (decay time)	120–570	2.08 (GAG)	Temperature sensing	[71]
			Lifetime (risetime)		3.58 (GGG)		
20–200	Sr_4_Al_14_O_25_	Mn^4+^ (Tb^3+^)	Ratiometric, (Mn^4+^/Tb^3+^ bands)	123–543	2.8	Thermal imaging	[72]
<100	Ba_3_(VO_4_)_2_	Mn^5+^ (Nd^3+^)	Ratiometric, (Nd^3+^/Mn^5+^ bands)	83–683	0.94	Deep-tissue thermal imaging	[73]
10–50	SrTiO_3_	Ni^2+^ (Er^3+^)	Ratiometric, (Ni^2+^ bands)	123–483	0.25	Temperature sensing in the second biological window	[74]
			Ratiometric (Er^3+^/Ni^2+^ bands)		0.80		

**Table 4:** The overview of the most recent literature reports on semiconductor quantum dots for luminescence temperature sensing.

QDs Size [nm]	Type of QDs	Measurement Method	Temperature Range [K]	Maximal Sensitivity [%K^−1^]	Application	Ref.
5–6	CdSe/CdS/ZnS dispersed in squalane	$\mathrm{Linear}\;\mathrm{dependence}\;E_{emission}-T$	295–393	NA	Temperature measurement in situ of thin films of liquids in dynamic conditions	[88]
~7.5	CdSe/ZnS immobilised in silicone matrix	$\mathrm{Linear}\;\mathrm{dependence}\;E_{emission}-T$	303–333	0.93	In situ thermal analysis and calibration within the microfluidics	[89]
~7.5	CsPbCl_3_:Mn^2+^	$\mathrm{Ratiometric}\;(I_{Mn}/I_{Exciton}$)	298–353	7.38	Highly sensitive temperature measurement of nanoprobes	[90]
		FWHM method (Exciton emission)		2.13		
~6	CdHgTe in NaCl matrix	$\mathrm{Linear}\;\mathrm{dependence}\;E_{emission}-T$	80–340	0.02	NIR LED, NIR noncontact thermometry	[91]
		Lifetime		1.4		
~4	PbS/CdS/ZnS	Intensity	283–333	1	Minimally invasive photothermal tumor treatments with real-time intratumoral thermal feedback	[92]
NA	CdSe/CdS_x_Se_1−x_	Ratiometric (FWHM and the maximum emission intensity)	306–343	6.9	Supersensitive luminescent nanothermometers with low toxicity	[93]

**Table 5:** The overview of the most recent literature reports on polymer nanoparticles for luminescence temperature sensing.

PNPs Size [nm]	Type of PNPs	Measurement Method	Temperature Range [K]	Maximal Sensitivity [% K^−1^]	Application	Ref.
20–50	NIR775 dye in MEH-PPV polymer	Lifetime of persistent luminescence	277–308	NA	Optical imaging in living mice	[98]
90	Rhodamine dye in F127-melamine-formaldehyde polymer	Ratiometric (R_110_ emission/R_B_ emission)	253–383	7.6	Cellular temperature monitoring in HeLa cells during microwave exposure	[99]
NA	Ru(II) polypyridyl complexes embedded in poly(cyanoacrylate)	Luminescence intensity (linear dependence I−T)	273–323	3.3	Production of temperature-sensitive optical fiber tips for temperature and O_2_ monitoring	[100]
~20	Eu^3+^,Sm^3+^-btfa-based polymeric micelles	Ratiometric, dual emission (Sm emission/Eu emission)	300–328	1.5	Intracellular temperature mapping of breast metastatic adenocarcinoma cells	[101]
	Eu^3+^,Sm^3+^-DNPD-based polymeric micelles			1.7		
NA (water solutions)	Tetrasulfonatophenylporphyrinate (tetra sodium salt) TSPH_2_	Ratiometric (peak-to-valley ratio)	293–318	1.04	Molecular thermometers for phototherapy to avoid heat-induced adverse effects	[102]
	Mono(4-pyridyl)-triphenylporphyrinato)phosphorus(V) bromide MPyPP(OH)_2_	Lifetime-based		0.24		
8	Poly(N-vinylcaprolactam)	Aggregation-induced emission after heating	293–313	NA	Intracellular temperature imaging in MCF-7 cells	[103]
NA (in solutions)	Green Fluorescent Protein	Peak Fractions analysis	293–333	2.5	Determination of chemically induced heat production in the HeLa cells	[104]
NA	Carbazole-substituted dicyanobenzenes, anthraquinone based dyes in RL100 nanoparticles	Lifetime-based	278–323	2.8	Temperature sensing and imaging	[105]
150 (178)	Nanocapsules consisting of PdPc(OBu)_8_, PCU, perylene, nujol (medium), albumin (stabilizer)	Lifetime-based	293–318	~7.5	Detection of the temperature changes in vivo in mice	[106]

**Table 6:** The overview of the most recent literature reports on carbon dots for luminescence temperature sensing.

CDs Size [nm]	Type of CDs	Measurement Method	Temperature Range [K]	Maximal Sensitivity [% K^−1^]	Application	Ref.
~4	CD	Linear dependence I−T(intensity rises with temperature)	283–353	1.07	Temperature measurement in living MC3T3-E1 cells	[123]
~2.3	N, S—CD	Linear dependence I−T	298–348	NA	Temperature sensor	[124]
~27	CD	Ratiometric, dual emission	288–358	0.93	Cellular temperature imaging of MC3T3-EI cells	[125]
~1.5	CD	Ratiometric, dual emission	278–358	1.48	Temperature sensor	[126]
~2.5	N, S—CD	Linear dependence I−T	278–348	0.41	Temperature sensor	[120]
~7.5	CD	Ratiometric, dual emission	278–333	3.71	In vitro thermal sensing using HeLa cells	[127]
~3	CD	Ratiometric (intensity rises with temperature)	277–353	1.2	Intracellular temperature sensing in different cells	[128]
~3.6	N, S—CD	Ratiometric	283–343	0.64	Temperature sensor	[129]
~5.6	MnO—CD	Ratiometric	283–333	NA	In vitro thermal sensing using HepG2 cells	[130]
<5	CD	Ratiometric, dual emission	305–315	8.2	Intracellular temperature measurement in HEK293T cells	[115]

**Table 7:** The overview of the most recent literature reports on nanodiamonds for luminescence temperature sensing.

NDs Size [nm]	Type of luminescent Center	Measurement Method	Temperature Range [K]	Maximal Sensitivity	Application	Ref.
100	NV	All-optical	85–300	~15 K/Hz^−1/2^	Low-temperature thermometry	[149]
100	NV	ODMR	RT	~2 K/Hz^−1/2^	Camera-based thermometry of living cells	[150]
100	NV	All-optical	RT	~0.2 K/Hz^−1/2^	Nanothermometry in the biological transparency window	[151]
	NiV	ODMR		~0.9 K/Hz^−1/2^		
40	NV	ODMR (frequency-jump modulation)	RT ∆T~20 K	NA	Thermal probing and heat diffusion measurements in the microelectronic circuit	[152]
100	NV	All-optical	301–348	2 K/Hz^−1/2^	Hyperthermia research in human embryonic kidney cells	[153]
168	NV	ODMR	303.4–317.3	1.4 K/Hz^−1/2^	Temperature measurement in living animals which may allow quantification of their biological activities	[154]
150	NV	ODMR	302.1–313.9	2.2 K/Hz^−1/2^	Measurement of the intracellular thermal conductivities of HeLa and MCF-7 cells	[155]
70	SiV	All-optical	RT	0.037 K^−1^	Temperature monitoring and mapping within intracellular regions	[144]
200	SiV	All-optical	RT ∆T~20 K	~0.5 K/Hz^−1/2^	Calibration-free thermometry for sensing and control of complex nanoscale systems	[156]
300	SiV	All-optical	200–400	~0.5 K/Hz^−1/2^	Practical nanoscale thermometry and sensing	[157]

## Data Availability

The manuscript does not contain any original data.
